# High prevalence of human papillomavirus (HPV) in oral mucosal lesions of patients at the Ambulatory of Oral Diagnosis of the Federal University of Sergipe, Northeastern Brazil

**DOI:** 10.1590/1678-77572016-0313

**Published:** 2017

**Authors:** Mariana Goveia Melo RIBEIRO, Larissa Doddi MARCOLINO, Bruna Ribeiro de Andrade RAMOS, Elaine Alves MIRANDA, Cleverson Luciano TRENTO, Sona JAIN, Ricardo Queiroz GURGEL, Márcia Guimarães da SILVA, Silvio Santana DOLABELLA

**Affiliations:** 1 Universidade Federal de Sergipe, Laboratório de Entomologia e Parasitologia Tropical, Departamento de Morfologia, São Cristóvão, SE, Brasil.; 2 Universidade Estadual Paulista, Faculdade de Medicina de Botucatu, Departamento de Patologia, Botucatu, SP, Brasil.; 3 Universidade Federal de Sergipe, Departamento de Odontologia, Aracaju, SE, Brasil.; 4 Universidade Federal de Sergipe, Departamento de Medicina, Aracaju, SE, Brasil.

**Keywords:** Papilomaviridae, Mouth mucosa, Epidemiology, Brazil

## Abstract

The role of human papillomavirus (HPV) in oral carcinogenesis is still controversial as detection rates of the virus in oral cavity reported in the literature varies greatly.

**Objective:**

The aim of this study was to evaluate the frequency of HPV infection and its genotypes in patients with oral lesions at the Ambulatory of Oral Diagnosis of the Federal University of Sergipe, Brazil.

**Material and Methods:**

We conducted a molecular study with 21 patients (15 females) aged from two to 83 years with clinically detectable oral lesions. Samples were collected through exfoliation of lesions and HPV-DNA was identified using MY09/11 and GP5+/6+ primers. Genotyping was performed by multiplex PCR.

**Results:**

Benign, premalignant and malignant lesions were diagnosed by histopathology. HPV was detected in 17 samples. Of these, HPV-6 was detected in 10 samples, HPV-18 in four and HPV-16 in one sample. When samples were categorized by lesion types, HPV was detected in two papilloma cases (2/3), five carcinomas (5/6), one hyperplasia (1/1) and nine dysplasia cases (9/11).

**Conclusion:**

Unlike other studies in the literature, we reported high occurrence of HPV in oral lesions. Further studies are required to enhance the comprehension of natural history of oral lesions.

## Introduction

Oral cancer is one of the most common types of cancer. In 2012 there were approximately 264 thousand new cases worldwide and 128 thousand people died due to this disease^9^. In Brazil oral cancer is among the ten most common cancers and is an important public health concern. According to recent estimates by the Brazilian National Institute of Cancer (INCA), oral cancer is the fourth more frequent type of cancer among men and the ninth among women in the Northeast region of Brazil^9^.

Chemical, physical and biological factors are associated with the development of oral lesions. Alcohol consumption and smoking habits are important factors for carcinogenesis and are associated with most cases^1^. Among the infectious agents related to oral cancer, HPV has been greatly implicated in the pathogenesis and worsening of lesions^2,23^. Recent data show that the presence of HPV and the aforementioned detrimental habits are independent etiological factors for oral cancer. Moreover, it has been suggested that oral cancer could be clustered according to these two etiological factors and that patients with HPV-induced cancers present better prognostic and survival rates than those with detrimental habits-induced cancer^13^.

Several genotypes of HPV can infect superior airways. Low-risk genotypes are mainly associated with benign lesions while high-risk genotypes are frequently associated with malignant lesions in oral cavity, especially squamous cell carcinoma (SCC)^2^. The detection rate of HPV in oral lesions varies significantly around the world, ranging from undetectable to 100% frequency. A systematic review evaluated over 60 studies that identified HPV in head and neck cancers by PCR. The authors described that 25.9% of the tumors had detectable HPV, 35.6% of those in the oropharynx and 23.5% in the oral cavity^19,21^. Nevertheless, the role of HPV in oral carcinogenesis is still controversial^2,16^.

Thus, this study aimed to evaluate the prevalence of HPV and their genotypes in patients with oral lesions at the Ambulatory of Oral Diagnosis of the Federal University of Sergipe, Brazil.

## Material and methods

### Patients

We conducted a molecular study with 21 convenience samples from men (six) and women (15) visiting the Ambulatory of Oral Diagnosis of the Federal University of Sergipe, Brazil, between January 2013 and March 2014. All the patients presented clinically detectable oral mucosa lesions. The study protocol was approved by the Research Ethics Committee of the Federal University of Sergipe (Protocol 76317). Informed consent form was obtained from all patients. Sociodemographic data were obtained from medical records and through standardized questionnaires.

Samples that did not fulfill the following criteria were excluded from the study: (i) absence of signed consent form at the time of collection; (ii) inability to collect the samples from the lesion; (iii) inadequate quality of the collected sample, that is, failure in the amplification of human β-globin gene by PCR.

### Sample collection and processing

Clinically detectable lesions were submitted to total or partially biopsy. Tissue samples were fixed in formalin and embedded in paraffin for staining with hematoxylin-eosin and microscopy analysis.

For HPV detection and genotyping, samples were collected from oral mucosa by exfoliating the lesions with sterile cytobrush before biopsy. Samples were stored in 70% ethanol at 4°C up to 15 days until processing.

### Molecular detection and characterization

DNA was extracted by enzymatic digestion using lysis solution containing proteinase K. Samples were centrifuged at 3500 rpm for 5 minutes and supernatants were discarded. Pellets were diluted in 500 µL of lysis solution (5M NaCl; 0.5M Tris-HCl pH7.6; 0.5M EDTA pH8; 10%SDS; 0.5 mg/mL Proteinase K) and transferred to 1.5 mL Eppendorf^®^ tubes for incubation at 60°C for 5 hours. Later, 67 µL of 5 M NaCl was added, tubes were vortexed vigorously for 30 seconds and centrifuged at 14000 rpm for 20 minutes at 4°C. Supernatants were then transferred to new tubes and 400 µL of cold isopropanol was added. Samples were incubated overnight at -20°C and centrifuged again at 14000 rpm for 20 minutes at 4°C. The tubes were washed with 400 µL of 70% ethanol and centrifuged at 14000 rpm for 10 minutes at 4°C. Extracted DNA was resuspended in 30 µL of Tris-EDTA and stored at -20°C until processing.

**Figure 1:**
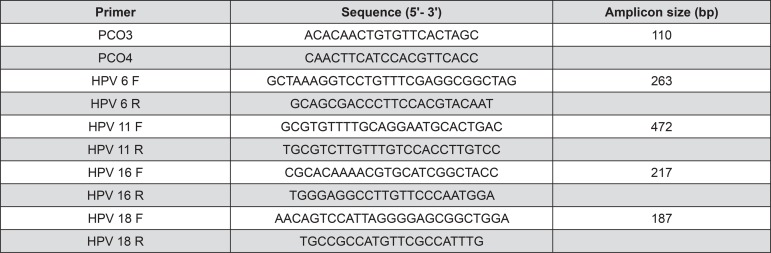
Primer sequences used for PCR

In order to check the integrity of extracted DNA and the absence of PCR inhibitors, a 110 bp segment of human β-globin gene was amplified using PCO3/PCO4 primers^6^. Sequences of primers are displayed in Figure 1. Amplified DNA was separated by electrophoresis in 2.0% agarose gel (Invitrogen, Carlsbad, CA, USA) stained with Gel Red^TM^ (New England BioLabs; Ipswich, MA, USA) at 100 V and 10 mA for 1 hour. Results were visualized by UV transillumination.

Detection of HPV sequences was performed by nested PCR using two sets of consensus primers, MY09/MY11 and GP5+/GP6+, which amplify a 450 bp fragment and an internal fragment of 150 bp, respectively, of the highly conserved *L1* HPV gene (9.15). PCR reactions were performed in Veriti^®^ Thermal Cycler (Applied Biosystems; Foster City, CA, USA) and contained 10 mL PCR Buffer Go Taq Green (Promega; Madison, WI, USA), 0.6 mL of each primer (10 mM), Milli-Q water (Milipore; Billerica, MA, USA) and 2 mL of each sample in a final volume of 20 mL. First, the 450 bp fragment was amplified using the following PCR conditions: initial denaturation for 5 minutes at 95°C; followed by 36 cycles of 1 minute at 94°C, 1 minute at 50°C and 1 minute at 72°C; and final extension at 72°C for 10 minutes. The second amplification targeting the internal region was performed with 2 mL of the amplicon and PCR conditions as follows: initial hold of 5 minutes at 95°C; 45 seconds at 95°C, 45 seconds at 47.7°C and 1 minute at 72°C for 44 cycles; and a final extension of 7 minutes at 72°C. Negative controls (sterile water) and HPV-16 positive controls extracted from HeLa cells were used in all reactions.

Genotyping was performed using multiplex PCR with specific sets of primers for genotypes 6, 11, 16 and 18 (Figure 1) using the same mix conditions as described above. Thermo cycling parameters used were: initial denaturation for 1 minute at 94°C; followed by 37 cycles of 1 minute at 94°C, 30 seconds at 53.5°C and 1 minute at 72°C; and a final extension at 72°C for 7 minutes.

### Statistical analysis

Comparisons of HPV frequency related to lesions type were made using Fisher test using the GraphPad Prism 5.0 software (GraphPad; La Jolla, CA, USA), considering p<0.05 to be statistically significant.

## Results

Sociodemographic characteristics of the participants are displayed in Table 1. The majority of patients were female (71.4%) and defined themselves as nonwhite (90.4%). Regarding educational status and health behaviors, 52.4% of patients were literate and 52.4% declared to regularly consume alcohol or tobacco.

**Table 1:** Sociodemographic data of the patients with oral mucosa lesions used in this study

Characteristics	N (%)	HPV occurrence
**Gender**		
Female	15 (71.4%)	12 (80%)
Male	06 (28.6%)	05 (83.33%)
**Ethnic group**		
White	02 (9.5%)	02 (100%)
Nonwhite	19 (90.4%)	15 (78.9%)
**Education**		
Literate	11 (52,4%)	09 (81.8%)
Nonliterate	10 (47.6%)	08 (80%)
**Age**		
Up to 50	06 (28.6%)	04 (66.6%)
50 to 65	09 (42.9%)	07 (77.7%)
65 to 83	06 (28.6%)	06 (100%)
**Smoking Status**		
Smoker	7 (33.3%)	06 (85.7%)
Nonsmoker	14 (66.6%)	10 (71.4%)
**Alcohol Use**		
Yes	6 (28.6%)	03 (50%)
No	15 (71.4%)	14 (93.3%)

The lesions were located throughout the hard palates (19.0%), floor of the mouth (4.8%), buccal mucosa (28.6%), lips (19.0%) and dorsal and ventral surfaces of the tongue (28.6%). HPV prevalence in collected samples was 81.0%. From this total, 35.3% of the HPV could not be genotyped with the techniques employed in the study. The most prevalent HPV type was the low-risk HPV-6 (58.8%), followed by high-risk HPV-18 (23.5%) and HPV-16 (5.9%). Multiple-type infections (HPV-6 and 18) were presented in 23.5% of patients with HPV. β-globin was detected in all samples evaluated.

Histopathologically, lesions were categorized as hyperplasia (4.8%), squamous papillomas (14.3%), squamous cell carcinoma (28.6%) or epithelial dysplasia (52.3%), and 33.3% of samples presented koilocytosis. As *per* histolopathological diagnosis, HPV was detected in 2/3 of papilloma cases, in 5/6 of carcinomas, in one case of hyperplasia and in 9/11 of cases with dysplasia. The statistical analysis between HPV genotype and histopathological diagnosis was performed and no association was found (p>0.165). HPV detection and genotyping according to the histopathological diagnosis of the lesions is shown in Table 2.

**Table 2:** Prevalence of HPV and type clustering according to histopathological diagnosis

Type of Lesion	HPV occurrence	HPV-6	HPV-16	HPV-6/18	Not genotyped
Hyperplasia	1/1				1
Papilloma	2/3	1	1		
SCC	5/6	3			2
Dysplasia	9/11	2		4	3
Total	17/21	6	1	4	6

SCC: Squamous cell carcinoma

No association was found between HPV genotype and histopathological diagnosis (p>0.165)

## Discussion

Apart from a lot of debate regarding the role of HPV as etiological agent in oral cancer development and worsening of the lesions, the mechanism of HPV transmission to oral cavity has also not been fully elucidated. Oral-genital contact has been considered the main route of HPV transmission to oral cavity^7,13^, although other theories include perinatal transmission, self-contamination and mouth-to-mouth transmission^5^. Although HPV has been detected in specimens of oral dysplasia and cancer, the prevalence of HPV and its role in the pathogenesis of dysplasia and cancer is still unclear^24^.

The present study detected HPV-DNA in 81.0% of clinically detectable lesions in oral mucosa, thus indicating a probable role HPV could play in oral lesions. This finding is in agreement with those observed by Morbini, et al.^15^ (2012) who identified the virus in 87.5% of SCC samples from head and neck cancers and dysplasia in Italy, and also Jordan, et al.^10^ (2012) who detected HPV in 78.3% of 233 samples with SCC in the United States.

However, our data differs from other studies performed in Brazil that report HPV rates ranging from 0% to 30% in oral lesions^1,14,22^. In São Paulo state, Rivero and Nunes^22^ (2006) could not identify HPV in any of the 40 samples with SCC. Using *in situ* hybridization, Acay, et al.^1^ (2008) detected HPV in 24% of samples with oral lesions with leukoplakia, dysplasia and SCC. Another study by Miyahara, et al.^14^ (2011) reported that 33.7% of 83 samples with SSC were positive for presence of HPV. Regarding the Northeast region of the country, few studies have evaluated viral presence in oral lesions so far. In a study carried out in Pernambuco state, Vidal, et al.^29^ (2004) detected HPV in 27.5% of samples with SCC using hybrid capture. To our knowledge, our study is the first to evaluate the presence of HPV in oral lesions in Sergipe state.

The high HPV index found in our study could be related to demographic issues inherent in our population, in which 90.4% of patients declared themselves as nonwhite. This is related to the finding of Tsao, et al.^28^ (2016), according to which the only significant association found was between nonwhite patients and a higher prevalence of HPV in oral swabs.

An interesting aspect of HPV prevalence in oral lesions is that it markedly varies worldwide, and even within the same country. For instance, in two studies performed in Italy that used similar techniques, Termine, et al.^27^ (2012) reported prevalence of 25.3% HPV cases while Morbini, et al.^15^ (2012) described a rate of 87.5%. It has been suggested that geographical variability influences HPV rates in oral lesions^21^. Moreover, distinct techniques used may also account for the difference in the rates reported in different studies, which interferes with comparisons^6,21^, and variability in evaluated lesions types.

Although the patients with HPV lesions are generally described as younger, nonsmokers and non-alcoholic, these data are still controversial. Our data, similar to the data of several authors, show no association between these factors and HPV status^20,24^. No significant difference was observed in the frequency of HPV infection between men and women, as also observed in the study by Tatar, et al.^26^ (2015). This is also in accordance with other studies that suggest that in some cultures both genders are exposed in a similar manner to risk factors for oral HPV infection^18^.

In order to enhance HPV detection we used the nested-PCR technique that uses more than one pair of primers. Thus, we were able to detect the virus even in very low concentrations. High quality DNA is required for this technique to reach its optimal conditions. Acay, et al.^1^ (2008) could not obtain the required amount of DNA from paraffin-embedded tissue samples and indicated that this may be the explanation for the low prevalence of HPV reported in studies with premalignant and malignant lesions using nested-PCR in that specimen type. Another advantage of our study is uniformity of material, sampling and processing procedures.

The presence of HPV reported in the literature is independent of malignant grading^14,27^. Presently, we evaluated malignant (SCC), premalignant (dysplasia) and benign (hyperplasia and papilloma) lesions and reported HPV infection in all types of lesions.

The presence of koilocytosis is considered to be the signature of HPV infections^14,29^. According to Hajdu, et al.^8^ (2006), HPV-DNA is present in 75% of lesions with koilocytosis in cervical samples. Similarly, in the present study we found 71.4% of HPV prevalence in samples with koilocytosis.

For genotyping, primers pertaining to the four most common HPV genotypes associated with head and neck cancer were utilized^4,12,13^. HPV-6 was the most frequent viral type in our samples, identified in 90.9% of the genotyped HPV samples. Bharti, et al.^3^ (2013) in their study showed the presence of this type in six out of nine oral lesions. Along with HPV-16, HPV-6 is one of most frequently reported types in oral cancer. Kristoffersen, et al.^12^ (2012) identified HPV-6 in 48% of premalignant lesions and Sugiyama, et al.^25^ (2003) detected HPV-16 in 61% and 35% of samples with dysplasia and SCC, respectively, and HPV-18 in only two samples with SCC (3.5%). Here we report prevalence of 23.5% for HPV-18 and 5.9% for HPV-16 in HPV-positive samples. We were not able to genotype 35.3% of our HPV-positive samples probably because these patients were infected with types different than what we tested for. Similar results have been reported by Mravak-Stipetic, et al.^17^ (2013) who could not genotype half of their HPV-positive samples.

Comparative studies concerning HPV infection sites have been performed around the world. Presence of HPV-DNA has been detected in healthy oral mucosa^7^ as in all types of oral lesions^11^. However, there are few studies so far, especially in the Northeast region, to evaluate HPV oral infection. The main limitation of our study was the small sample size, which prevented a more robust statistical analysis. Another limitation was the lack of data on sexual behavior of patients, which could help understand the route of viral transmission.

The HPV vaccination is currently available in several countries, including Brazil, where the quadrivalent vaccine is distributed by the government to female preadolescents aged 11 to 13 since March 2014. Although the importance of vaccination in reducing genital infections is well documented, the effect of the vaccine in preventing head and neck lesions has not been addressed yet^30^. It is possible that the rate of oral cancer in vaccinated women will be reduced in the future, which could contribute to the understanding of the role of HPV in this disease^3^.

## Conclusions

Similarly to other studies, we report high occurrence of HPV in oral lesion. The results are of special importance, since they are one of the few from the Northeast Brazil and the first from the state of Sergipe. More data on this field is needed to improve comprehension of HPV distribution in Sergipe and in Brazil as a whole. Moreover, even though HPV seems to play an important role in oral carcinogenesis, studies that evaluate the association of HPV with normal oral mucosa are crucial to improve the comprehension of the role of HPV in oral lesions.
